# *YGL138*(*t*), encoding a putative signal recognition particle 54 kDa protein, is involved in chloroplast development of rice

**DOI:** 10.1186/1939-8433-6-7

**Published:** 2013-03-27

**Authors:** Fantao Zhang, Xiangdong Luo, Biaolin Hu, Yong Wan, Jiankun Xie

**Affiliations:** College of Life Sciences, Jiangxi Normal University, Nanchang, 330022 China; Biotechnology Research Institute, Jiangxi Academy of Agricultural Sciences, Nanchang, 330200 China

**Keywords:** *Oryza sativa* L, Yellow-green leaf mutant, Chloroplast development, SRP54 protein

## Abstract

**Background:**

Normal development of chloroplast is vitally important to plants, but its biological mechanism is still far from fully being understood, especially in rice.

**Results:**

In this study, a novel yellow-green leaf mutant, *ygl138*, derived from Nipponbare (*Oryza sativa* L. ssp. *japonica*) treated by ethyl methanesulfonate (EMS), was isolated. The mutant exhibited a distinct yellow-green leaf phenotype throughout development, reduced chlorophyll level, and arrested chloroplast development. The phenotype of the *ygl138* mutant was caused by a single nuclear gene, which was tentatively designed as *YGL138*(*t*). The *YGL138*(*t*) locus was mapped to chromosome 11 and isolated into a confined region of 91.8 kb by map-based cloning. Sequencing analysis revealed that, *Os11g05552*, which was predicted to encode a signal recognition particle 54 kDa (SRP54) protein and act as a chloroplast precursor, had 18 bp nucleotides deletion in the coding region of *ygl138* and led to a frameshift. Furthermore, the identity of *Os11g05552* was verified by transgenic complementation.

**Conclusions:**

These results are very valuable for further study on *YGL138*(*t*) gene and illuminating the mechanism of SRP54 protein involving in chloroplast development of rice.

**Electronic supplementary material:**

The online version of this article (doi:10.1186/1939-8433-6-7) contains supplementary material, which is available to authorized users.

## Background

On our globe, up to 10^9^ tons of chlorophyll is synthesized and degraded every year (Eckhardt et al. 2004). In higher plants, the content of chlorophyll in chloroplast is more than that of other pigments. Its significant change always results in the variation of leaf color, so chlorophyll deficient mutation is also called leaf-color mutation.

The mechanism of leaf-color mutation is very complicated. As an excellent material in such studies, leaf-color mutants have been paid more and more attentions recently (Jung et al. 2003; Zhang et al. 2006; Wu et al. 2007). During the past decade, large number of such mutants had been identified in higher plants, such as rice (Hu et al. 1981), soybean (Ghirardi and Melis 1988), wheat (Falbel et al. 1996), Arabidopsis (Carol et al. 1999), maize (Pasini et al. 2005), and barley (Liu et al. 2008(Nagata et al.). At the same time, there had been major advancements in understanding genetic mechanism of leaf-color mutation, with many related genes being identified. So far, genes for all 15 steps in the chlorophyll biosynthetic have been identified in Arabidopsis. Analysis of the complete genome of Arabidopsis indicated that it has 15 enzymes encoded by 27 genes for chlorophyll biosynthesis from glutamyl-tRNA to Chl *b*^2005^).

Rice is an important food crop and a model monocotyledonous plant. At least 70 leaf-color mutants have been identified in it (Kurata et al. 2005). However, its mechanism of leaf-color mutation is still far from fully being understood, and only a few related genes have been cloned up to now. *OsCHLH* encodes OsChlH subunit of magnesium chelatase, and its mutant lacks chlorophyll in the thylakoids (Jung et al. 2003; Goh et al. 2004). *ygl1* encodes a chlorophyll synthase, and its mutant can reduce chlorophyll accumulation and delay chloroplast development (Wu et al. 2007). *Chl1* and *Chl9* encode key enzymes for chlorophyll synthesis and chloroplast development. Ultrastructural analyses have revealed that the grana of *Chl1* and *Chl9* mutants are poorly stacked, resulting in the underdevelopment of chloroplasts (Zhang et al. 2006). *OsCAO1* and *OsCAO2* encode Chl *a* oxygenase. *OsCAO1* is induced by light; in contrast, *OsCAO2* mRNA expression level is higher under dark condition (Lee et al. 2005). *OsDVR* encodes a functional 8-vinyl reductase and can convert divinyl Chl *a* to monovinyl Chl *a* in rice, and its mutant exhibits a yellow-green leaf phenotype (Wang et al. 2010). *OsPPR1* encodes the PPR protein that might play an essential role in the chloroplast biogenesis, and its mutant shows typical phenotypes of chlorophyll-deficient mutant (Gothandam et al. 2005). *OsHAP3* regulates the normal development of chloroplasts and chlorophyll biosynthesis, and its mutant shows pale green leaves (Miyoshi et al. 2003). *RNRL1* and *RNRS1* encode the subunits of ribonucleotide reductase that is required for chloroplast biogenesis during early leaf development, and their mutants exhibit chlorotic leaves in a growth stage-dependent manner under field condition (Yoo et al. 2009).

Here, we identified a novel yellow-green leaf mutant *ygl138*, which was controlled by a single nuclear gene in rice. We tentatively named it *YGL138*(*t*). Map-based cloning of the mutation resulted in the identification of the *YGL138*(*t*) gene. *Os11g05552*, which was predicted to encode a signal recognition particle 54 kDa (SRP54) protein and act as a chloroplast precursor, had 18 bp nucleotides deletion in the coding region of *ygl138* and led to a frameshift. The mutant phenotype was complemented by transformation with the wild-type gene. Taken together, this study reports the identification of a SRP54 protein involving in the chloroplast development of rice.

## Results

### The *ygl138* mutant has reduced chlorophyll accumulation and arrested chloroplast development

**Figure 1:**
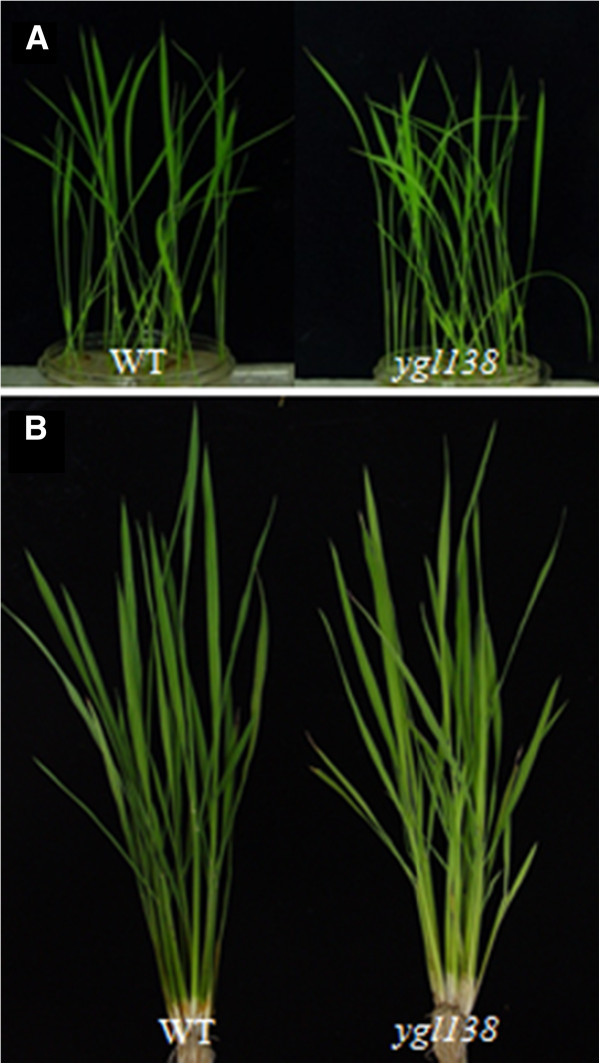
**Plant phenotypes of the** ***ygl138*** **mutant and its wild-type parent.** (**A**) Seedling stage. (**B**) Elongation stage. The *ygl138* mutant exhibits yellow-green leaf phenotype throughout development.

The *ygl138* mutant was derived from *japonica* rice cv Nipponbare treated by EMS, which exhibited a distinct yellow-green leaf phenotype throughout development (Figure 1). At the maturity, the tiller number, number of spikelets per panicle and seed setting rate reduced by 9.7%, 7.4%, and 9.6% respectively; but the plant height, panicle length, and 1000-grain weight were not affected remarkably compared with its wild-type parent (Table 1).

**Table 1:** **Comparison of major agronomic traits between the** ***ygl138*** **mutant and its wild-type parent**

Agronomic traits	WT	***ygl138***	Compared with WT (%)
Plant height (cm)	106.3 ± 3.8	102.2 ± 2.3	−3.9
Tiller number	12.4 ± 1.5	11.2 ± 1.7	−9.7^★^
Panicle length (cm)	19.5 ± 0.9	19.1 ± 1.2	−2.1
No. of spikelets per panicle	115.9 ± 5.7	107.3 ± 4.8	−7.4^★^
Seed setting rate (%)	91.3 ± 1.3	82.5 ± 1.9	−9.6^★^
1000-grain weight (g)	23.3 ± 0.3	22.9 ± 0.4	−1.7

^★^ Significantly different at *P* = 0.05.

To characterize the yellow-green leaf phenotype of the *ygl138* mutant, we measured its chlorophyll contents at the seedling stage. Compared with that of its wild-type parent, the content of Chl *a*, Chl *b* and total Chls decreased significantly in the mutant, indicating that the mutant phenotype resulted from reduced chlorophyll level. In addition, the ratio of Chl *a*/*b* was increased, due likely to the potential of Chl *b* synthesis in suffering a more severe decline than Chl *a* in the *ygl138* mutant (Table 2).

**Table 2:** **Comparison of chlorophyll contents in leaves between the wild-type parent and** ***ygl138*** **mutant at the seedling stage**

Line	Total Chl	Chl ***a***	Chl ***b***	Chl ***a***/***b***
	(mg/g FW)	(mg/g FW)	(mg/g FW)	Ratio
WT	3.01 ± 0.17	2.48 ± 0.16	0.53 ± 0.09	4.68 ± 0.11
*ygl138*	1.78 ± 0.08	1.57 ± 0.11	0.21 ± 0.04	7.48 ± 0.24
Compared with WT	−40.9%^★★^	−36.7%^★★^	−60.4%^★★^	59.8%^★★^

^★★^ Significantly different at *P* = 0.01.

**Figure 2:**
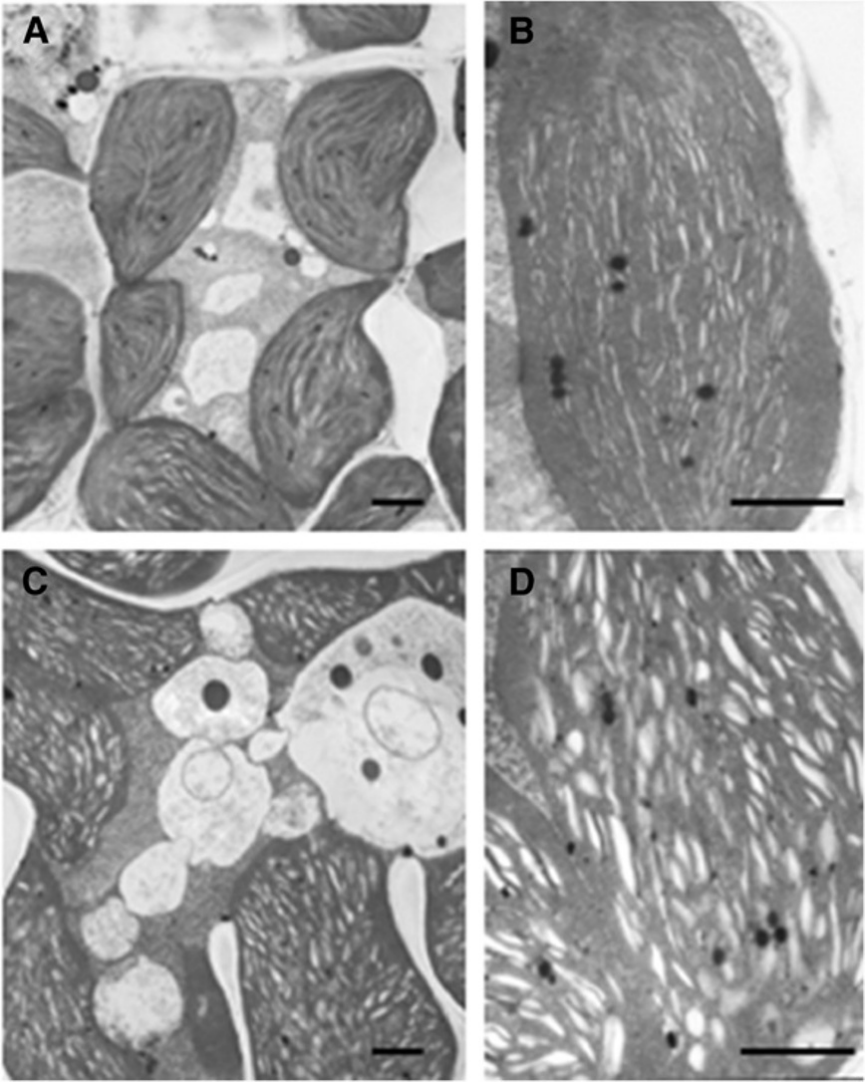
**Mesophyll cell structures of the mutant** ***ygl138*** **and the wild-type parent.** (**A**) Mesophyll cell of the wild-type parent. (**B**) Chloroplast structure of the wild-type parent. (**C**) Mesophyll cell of the *ygl138* mutant. (**D**) Chloroplast structure of the *ygl138* mutant. Bars indicate 1 μm in each panel.

**Figure 3:**
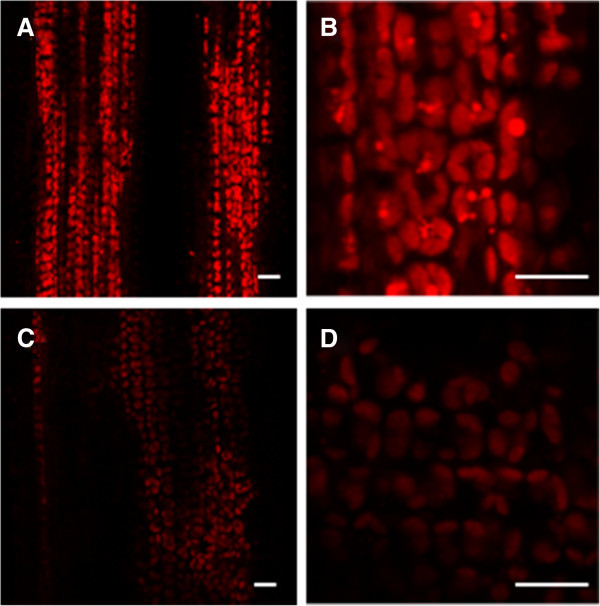
**Confocal laser scanning micrographs of mesophyll cells.** (**A, B**) The wild-type parent. (**C, D**) The *ygl138* mutant. The chloroplasts of *ygl138* mutant emitted a weaker chlorophyll fluorescence compared with wild-type chloroplasts. A and C indicate low-magnification, B and D indicate high-magnification. Bars indicate 10 μm in each panel.

Next, transmission electron microscopic analysis was performed to determine if low chlorophyll content in the *ygl138* mutant leaves affects the chloroplast development and morphology. The results revealed that, the number and size of chloroplasts per cell in the mutant were very similar to that of the wild-type parent (Figures 2A and C), whereas the shape of *ygl138* mutant chloroplasts was irregular compared with the wild-type chloroplasts, a lot of cystic structures were observed in the chloroplasts of *ygl138*, the number of thylakoids in *ygl138* was decreased, and the grana stacks in the mutant appeared less dense (Figures 2B and D). Furthermore, we used confocal microscopy to view the mesophyll cells of the leaf tissues, and the results revealed that the chloroplasts of *ygl138* mutant emitted a weaker chlorophyll fluorescence, compared with wild-type chloroplasts (Figure 3). These results indicated that the development of chloroplast was defective in the mutant.

### The *YGL138*(*t*) gene was fine mapped to a confined region about 91.8 kb on chromosome 11

To decipher the inheritance behavior of *ygl138* mutant, we developed an F_2_ population between *ygl138* and cultivar rice Minghui 63 and found that the F_1_ generation exhibited normal green leaf phenotype. Among the 3246 F_2_ individuals, 2410 were wild-type phenotype plants and 836 were yellow-green leaf phenotype plants. The separate ratio was about 2.88:1, which accorded with 3:1 (*χ*^2^ =1.0 < *χ*^2^_0.05, 1_ = 3.84). These results indicated that the yellow-green leaf phenotype of *ygl138* was controlled by a single nuclear gene, which was tentatively designed as *YGL138*(*t*).

**Figure 4:**
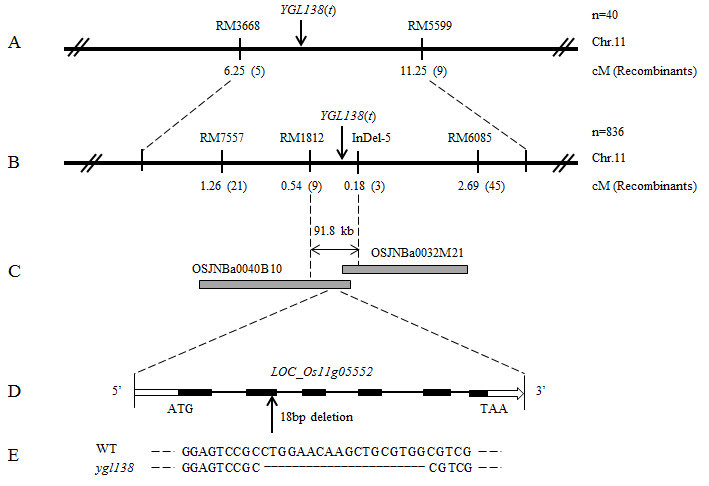
**Map-based cloning of** ***YGL138*** **(** ***t*** **).** (**A**) Primary mapping of *YGL138*(*t*). The *YGL138*(*t*) locus was primarily mapped between SSR markers RM3668 and RM5599 on the short arm of chromosome 11 with genetic distances 6.25 cM and 11.25 cM respectively. (**B**) Fine mapping of *YGL138*(*t*). The *YGL138*(*t*) gene was fine mapped between markers RM1812 and InDel-5 with the physical distance of 91.8 kb. (**C**) BAC contigs spanning the *YGL138*(*t*) locus. (**D**) Structure of *Os11g05552*. Black boxes indicate exons, white boxes indicate UTRs, and black lines indicate introns. (**E**) The mutation in the *ygl138* mutant. 18 bp nucleotides were deleted in the coding region of *Os11g05552* in the *ygl138* mutant.

In order to map the *YGL138*(*t*) gene, map-based cloning was carried out by using F_2_ population obtained from the cross of *ygl138* and Minghui 63. By screening 280 pairs of SSR markers scattered on rice chromosomes with proportional spacing, we found 105 pairs of markers exhibited polymorphisms between the two parents, and then these markers were used for analyzing the linkage relationship with the *YGL138*(*t*) gene. The yellow-green leaf mutant DNA pool and wild-type phenotype DNA pool were used for linkage relationship analysis. Finally, we detected that RM3668 and RM5599 on the short arm of chromosome 11 showed a linkage relationship with the *YGL138*(*t*) gene, and the *YGL138*(*t*) locus was primarily mapped between SSR markers RM3668 and RM5599 with genetic distances 6.25 cM and 11.25 cM, respectively (Figure 4A).

In the region between RM3668 and RM5599, 15 pairs of new SSR and InDel markers were developed for fine mapping. Four pairs of them exhibited polymorphism between two parents, and then were used for fine mapping (Additional file 1: Table S1). Finally, the *YGL138*(*t*) gene was mapped between markers RM1812 and InDel-5 based on 836 F_2_ recessive individuals from *ygl138*/Minghui 63. The genetic distances were 0.54 and 0.18 cM respectively, and the physical distance was about 91.8 kb (Figures 4B and C).

### Candidate gene annotation and sequence analysis

Within the 91.8 kb interval, no gene involved in leaf-color mutation has been cloned yet, and 10 predicted open reading frames (ORFs) were found (Table 3). To identify the candidate gene of *YGL138*(*t*), we sequenced the 10 ORFs in the *ygl138* mutant and its wild-type parent, respectively. DNA sequencing results revealed that 18 bp nucleotides were deleted in the coding region of *Os11g05552* in *ygl138* mutant (Figures 4D and E), and no nucleotide mutation existed in the other ORFs. The 18 bp were deleted at nucleotides 246 to 263 in the ORF in the *ygl138* mutant and caused a frameshift. Therefore, the *Os11g05552* gene was considered as the candidate gene of *YGL138*(*t*).

**Table 3:** **Gene names and their putative functions in the target interval**

Gene name	Putative functions
*Os11g05510*	carbonic anhydrase family protein, putative
*Os11g05520*	bifunctional monodehydroascorbate reductase and carbonic anhydrasenectarin-3 precursor, putative, expressed
*Os11g05530*	expressed protein
*Os11g05540*	rhoGAP domain containing protein, expressed
*Os11g05550*	expressed protein
*Os11g05552*	signal recognition particle 54 kDa protein, chloroplast precursor, putative, expressed
*Os11g05556*	signal recognition particle 54 kDa protein, putative, expressed
*Os11g05562*	40S ribosomal protein S25, putative, expressed
*Os11g05570*	RNASEH2A − Putative ribonuclease H2 large subunit, expressed
*Os11g05580*	conserved hypothetical protein

**Figure 5:**
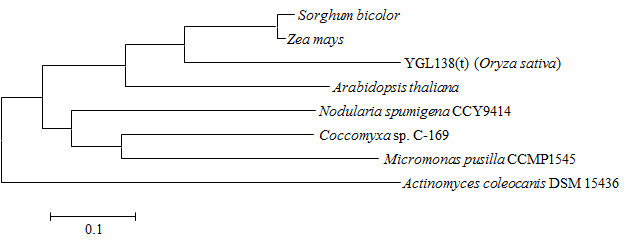
**A phylogenetic tree representing alignment of YGL138(t) protein and its homologues proteins.** The rooted neighbor-joining tree using percentage identities was constructed based on a multiple sequence alignment generated with the program Clustal X and MEGA 4. Scale represents percentage substitution per site. Accession numbers of the protein sequences were as follows: *Oryza sativa* [*YGL138*(*t*), RAPDB: *Os11g05552*]; *Coccomyxa* sp. C-169 [GenBank: EIE18517]; *Nodularia spumigena* CCY9414 [GenBank: ZP_01629394]; *Actinomyces coleocanis* DSM 15436 [GenBank: ZP_03925628]; *Arabidopsis thaliana* [GenBank: NP_196014]; *Micromonas pusilla* CCMP1545 [GenBank: XP_003056150]; *Sorghum bicolor* [GenBank: XP_002442824]; *Zea mays* [GenBank: NP_001142003].

BLAST search in the rice genome database revealed that *YGL138*(*t*) is a single-copy gene, and was predicted to encode a SRP54 protein and act as a chloroplast precursor. Multiple amino acid sequence alignment showed that YGL138(t) has significant similarity to *Zea mays* uncharacterized protein and *Sorghum bicolor* hypothetical protein, with identity of 80% and 75%, respectively. And its C-terminal region is poorly conserved from the homologues proteins of other species (Additional file 2: Figure S1). We subsequently analyzed the possible phylogenetic relationships between YGL138(t) and its related proteins from other species. The result indicated that rice YGL138(t) is more closely related to the homologues proteins of the monocotyledonous plants *Zea mays* and *Sorghum bicolor* than to those of other species (Figure 5).

### Complementation analysis

**Figure 6:**
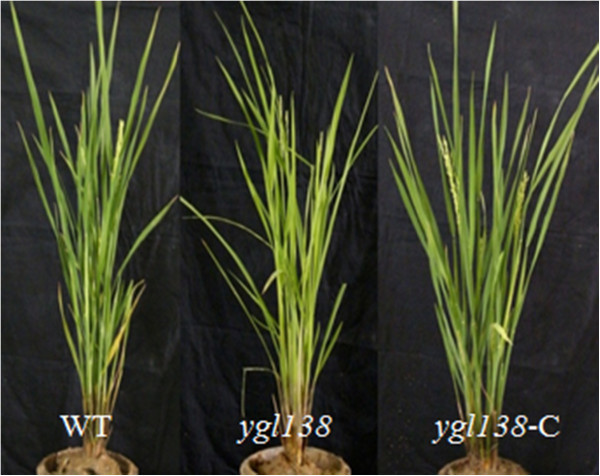
**Complementation analysis of the** ***ygl138*** **mutant.** Phenotype comparisons among wild-type, *ygl138* mutant and transgenic plants.

To confirm the defect of *Os11g05552* as causal mutation for *ygl138*, we performed a complementation analysis by transforming the wild-type gene (*Os11g05552*) into the *ygl138* mutant. Detected by PCR amplification, we obtained 20 positive transgenic plants. In these transgenic plants, the yellow-green leaf trait of *ygl138* was rescued (Figure 6). We measured their chlorophyll contents at the elongation stage, the results revealed that the levels of Chl *a*, Chl *b* and the ratio of Chl *a*/*b* in the transgenic plants were all restored to the levels of wild-type plant (Table 4). Furthermore, the other major traits of the transgenic plants were all restored to the normal levels (Table 5). These results confirm that the nucleotide deletion in *Os11g05552* was responsible for the *ygl138* mutant phenotype, and *Os11g05552* was identified as the *YGL138*(*t*) gene.

**Table 4:** **Comparison of chlorophyll contents in leaves among the wild-type parent,** ***ygl138*** **mutant and the transgenic plant at the elongation stage**

Line	Total Chl	Chl ***a***	Chl ***b***	Chl ***a***/***b***
	(mg/g FW)	(mg/g FW)	(mg/g FW)	Ratio
WT	5.32 ± 0.23	4.18 ± 0.20	1.14 ± 0.05	3.67 ± 0.13
*ygl138*	2.30 ± 0.13	1.93 ± 0.14	0.37 ± 0.02	5.22 ± 0.30
*ygl138*-C	5.28 ± 0.22	4.16 ± 0.30	1.12 ± 0.12	3.71 ± 0.18

**Table 5:** **Comparison of major agronomic traits among the** ***ygl138*** **mutant, its wild-type parent and the transgenic plants**

Agronomic traits	WT	***ygl138***	***ygl138***-C
Plant height (cm)	106.3 ± 3.8	102.2 ± 2.3	105.9 ± 2.4
Tiller number	12.4 ± 1.5	11.2 ± 1.7	12.5 ± 1.3
Panicle length (cm)	19.5 ± 0.9	19.1 ± 1.2	19.3 ± 0.6
No. of spikelets per panicle	115.9 ± 5.7	107.3 ± 4.8	114.8 ± 3.6
Seed setting rate (%)	91.3 ± 1.3	82.5 ± 1.9	90.8 ± 1.5
1000-grain weight (g)	23.3 ± 0.3	22.9 ± 0.4	23.1 ± 0.2

### *YGL138*(*t*) mRNA expression level is affected by the *YGL138*(*t*) mutation

**Figure 7:**
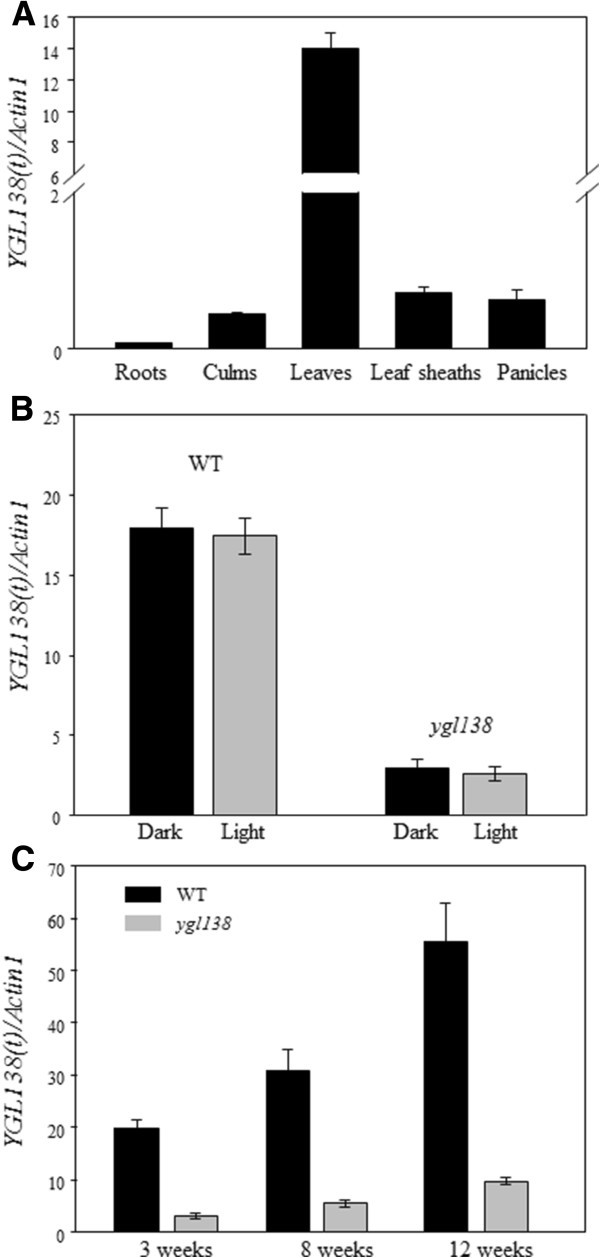
**Expression analysis of** ***YGL138*** **(** ***t*** **) by qRT-PCR.** (**A**) Expression patterns of *YGL138*(*t*) in different organs. (**B**) *YGL138*(*t*) expression in wild-type and the *ygl138* mutant leaves of 2-week-old plants grown in dark or under light. (**C**) *YGL138*(*t*) expression in wild-type and *ygl138* mutant leaves of 3-, 8-, and 12-week-old plants.

To investigate the *YGL138*(*t*) expression profile, total RNA was extracted from roots, culms, leaves, leaf sheaths, and panicles, and qRT-PCR analyses were performed. The results showed that *YGL138*(*t*) mRNA was expressed in all tissues tested, with relatively higher levels in leaves than in other organs (Figure 7A). We also examined the effect of light and dark growth conditions on the expression of *YGL138*(*t*). No change in transcript levels was observed when *ygl138* or wild-type plants were grown under light or dark conditions (Figure 7B). Furthermore, the transcript levels of *YGL138*(*t*) were always significantly decreased in *ygl138* than that of wild-type parents from early to mature stages (Figure 7C). These results indicated that the mRNA expression of *YGL138*(*t*) does not be affected by light intensity, and the mutation occurred in the coding region of *YGL138*(*t*) could result in the variation of its mRNA expression.

## Discussion

Nowadays, extensive attention has been paid to the leaf-color mutation, and certain achievements have been made by studying various organisms, but the mechanism of mutation and the responsible loci have not been fully understood at molecular level, especially in rice. Mutant analysis is a useful approach to illuminate the function of gene in complex biological processes of chloroplast development.

Recently, many reviews about *Arabidopsis* pigment defective mutants had been reported. *immutans* (*im*), *variegated1* (*var1*) and *variegated2* (*var2*) are the typical leaf variegation mutants, and exhibit green- and white-sectored leaves (Yu et al. 2007; Aluru et al. 2006; Sakamoto et al. 2003). However, in this study, we identified a novel yellow-green leaf mutant *ygl138* in rice, and defined it as a homogeneous paleness mutant.

By map-based cloning, the *YGL138*(*t* (Satoh et al.) gene was finally delimited in a 91.8 kb region on the short arm of chromosome 11. On the same chromosome, six genes: *V9*^1984^(Dong et al.); *tsc1*^2001^(Gothandam et al.); *OsPPR1*^2005^(Zhang et al.); *Z1*^2008^(Chai et al.); *Z2*^2011^(Liu et al.) and *yl11*^2012^), related to the leaf-color mutation have been mapped up to now, but only *OsPPR1* gene has been cloned. Among them, *V9* and *OsPPR1* mutants exhibit almost purely white at their young seedling stage, and leaves emerging at or after transplanting are pale green with white midrib (Satoh et al. 1984; Gothandam et al. 2005); *tsc1* mutant is a thermo-sensitive seedling-color mutant, and its thermo-sensitive decreases gradually with the increase of the seedling age (Dong et al. 2001); *Z1* mutants exhibit zebra leaves (Zhang et al. and *Z2*^2008^; Chai et al. 2011); *yl11* mutant is a typical chlorophyll deficient mutant, which has yellow leaves at whole growth stages (Liu et al. 2012). According to the previous reports, the six genes do not locate in the fine mapping region of *YGL138*(*t*), so we claim that *YGL138*(*t*) is a novel gene leading to leaf-color mutation in rice.

By sequencing, we found that, *Os11g05552*, which was predicted to encode a SRP54 protein and act as a chloroplast precursor, had 18 bp nucleotides deletion in the coding region of *ygl138* and led to a frameshift. Furthermore, the mutant phenotype of *ygl138* could be restored to the wild-type phenotype by transformation with the wild-type gene. Therefore, the gene *Os11g05552* was identified as the *YGL138*(*t*) gene.

In higher plants, chloroplast is a complex organelle with a highly organized internal system, and the chloroplast proteome comprises nuclear- and plastome-encoded proteins (Ferro et al. 2010). In order to function correctly, these proteins must be transported to their final destination in the chloroplast (Richter et al. 2010). In this progress, signal recognition particle (SRP) plays an essential role in binding to the signal sequence of the nascent polypeptide emerging from the ribosome and directing the polypeptide toward the proper cellular compartment (Keenan et al. 2001; Halic et al. 2004).

In eukaryotes, the SRP contains six proteins (SRP9, SRP14, SRP19, SRP54, SRP68, SRP72) and a 300-nucleotide RNA (Zwieb et al. 2005). Among them, SRP54 is the only protein subunit conserved in all SRPs, and it is essential for signal sequence recognition and binding at the ribosome, and for the GTP-dependent interaction with the cognate receptor SR. This interaction determines proper transport of the ribosome-nascent chain complex to the protein-translocating channel in the membrane (Egea et al. 2008). SRP54 can also be involved in the co-translational transport of chloroplast-encoded thylakoid proteins, which is able to switch between the co- and post-translational means of interaction with its respective substrate proteins (Franklin and Hoffman 1993; Li et al. 1995; Schünemann 2004). Meanwhile, the conserved SRP54 protein associates with 70S ribosomes to function in the co-translational transport of the plastid-encoded thylakoid membrane protein D1 (Nilsson et al. 1999; Richter et al. 2010). From these reports we can see that SRP54 is a key component in SRP protein and plays prominent roles in chloroplast development.

SRP54 is a basic protein and has a three-domain structure: an N-terminal helical bundle domain, a central GTPase domain and a C-terminal M domain (Marchler-Bauer et al. 2011). The N-terminal domain and the GTPase domain always associate together to constitute a structural and functional catalytic core, while the C-terminal M domain is responsible for the promiscuous recognition of the diverse signal sequences, and this domain is generally less conversed between different SRP homologues and also varies in length (Egea et al. 2008).

Mutant in SRP54 had been isolated in the model dicotyledonous plant Arabidopsis. *ffc1-2*, which was generated by EMS mutagenesis, always showed pale green leaves phenotype (Amin et al. 1999). This phenomenon was consistent with the reduction of chlorophyll levels in *ffc1-2* mutant compared with its wild-type plants (Hutin et al. 2002). However, to our knowledge, no SRP54 has yet been identified in model monocotyledonous plant rice. In our study, *Os11g05552*, which was predicted to encode a SRP54 protein and act as a chloroplast precursor, was identified as the novel gene *YGL138*(*t*) leading to leaf-color mutation in rice. We believe that further efforts on functional characterization of the *YGL138*(*t*) gene will contribute to understand the mechanism of SRP54 protein involving in chloroplast development in rice.

## Conclusions

A novel rice yellow-green leaf mutant *ygl138* was isolated in this study. The mutant exhibited a distinct yellow-green leaf phenotype throughout development. The phenotype of the *ygl138* mutant was controlled by a single nuclear gene, which was tentatively designed as *YGL138*(*t*). The *YGL138*(*t*) locus was fine mapped to a 91.8 kb region. A SRP54 gene (*Os11g05552*) was confirmed as the candidate gene by complementation analysis. These results will not only provide basis for further research on *YGL138*(*t*) gene, but will also facilitate the illumination of the mechanism of SRP54 protein involving in chloroplast development of rice.

## Methods

### Plant materials

The *ygl138* mutant was derived from Nipponbare treated by ethyl methanesulfonate (EMS). To fine map *YGL138*(*t*) gene, a large F_2_ population derived from the cross of *ygl138* mutant and normal green leaf variety Minghui 63 (*Oryza sativa* L. ssp. *indica*) was constructed, and 836 recessive individuals with yellow-green leaf phenotype in the population were selected for fine mapping. All the plants were grown under natural condition in a paddy field at the experimental station of Jiangxi Academy of Agricultural Sciences, Nanchang, China.

### Chlorophyll content measurement

Chlorophylls were extracted from 0.2 g fresh leaves with 95% ethanol, and the chlorophyll contents were measured with UV-1700 UV-visible spectrophotometer (Shimadzu, http://www.shimadzu.com/) and calculated according to the method of (Wellburn 1994).

### Transmission electron microscopy analysis

Fresh leaf sections of *ygl138* mutant and its wild-type parent Nipponbare were fixed in a solution of 3% glutaraldehyde and further fixed with 1% OsO4. The tissues were dehydrated in a gradient acetone series and embedded in Epon812 medium prior to thin sectioning. The samples were stained with uranyl acetate and Reynolds’ lead citrate, and observed under a transmission electron microscope (H-600IV, Hitachi, http://www.hitachi.com/).

### Confocal microscopy

Chlorophyll imaging was carried out using a confocal laser-scanning microscope (μRadiance; Bio-Rad Laboratories Inc., Hercules, CA, USA). Chlorophyll was excited with the 568 nm line. Fluorescence images were collected through red channel.

### Molecular marker development

Simple sequence repeats (SSR) markers were obtained from Gramene (http://www.gramene.org/microsat/) based on the SSR linkage map constructed by (McCouch et al. 2002). Insertion/Deletion (InDel) markers were developed according to DNA polymorphism of the mapped region between the *japonica* rice cv Nipponbare and the *indica* rice cv 9311 (http://www.ncbi.nlm.nih.gov/BLAST/). All InDel markers were designed by Primer 5.0 software.

### Mapping and linkage map

To screen the markers linkage to the *YGL138*(*t*) gene, two DNA pools were constructed from F_2_ population. One was mixed with 40 individuals with the yellow-green leaf phenotype of *ygl138*, and the other contained 40 individuals with wild-type phenotype. The markers covering the 12 chromosomes of rice were screened in two parents. 836 individuals with yellow-green leaf phenotype in F_2_ population were used for fine mapping. The PCR products of SSR and InDel markers were run on either 3% agarose gel or 8% polyacrylamide gel. According to the segregation data, molecular data were analyzed using MAPMAKER/EXP 3.0b software (Lander et al. 1987), and linkage map was constructed with MAPDRAW 2.1 (Liu and Meng 2003).

### Gene cloning and sequence analysis

The speculated candidate genes were amplified from *ygl138* mutant and its wild-type parent Nipponbare. Specific primers were designed according to the genome sequence of Nipponbare. And the primers for gene cloning and sequence analysis of *Os11g05552* were as follows: P1 (F: 5^′^-CCAGATAGTTTTGGGCCTGA-3^′^ and R: 5^′^-GCAGGCAATGCACAGAACTA-3^′^); P2 (F: 5^′^-GTCGGTGTACCAGTTTACTC-3^′^ and R: 5^′^-ACCAACGGGGAGGATAAGAG-3^′^). Target DNA fragments were cut from the gel and purified by the DNA Gel Extraction Kit (Tiangen, http://www.tiangen.com/), and then were sequenced at Shanghai Invitrogen Biotech. Co. Ltd. (Invitrogen, http://www.invitrogen.com/). Sequences data were assembled using ContigExpress, and then aligned using public programs Clustal X and Bioedit.

### Vector construction and transformation

For complementation of *ygl138* mutant, a 7179 bp genomic fragment consisting of a 2686 bp upstream sequence, the entire *YGL138*(*t*) coding region, and a 2284 bp downstream region was acquired by splicing together three DNA fragments from PCR amplifications. The primers used were as follows: CP1 (F: 5^′^-GGTACCGCTCATTGATTGGCTCTT-3^′^ and R: 5^′^-GGTTTACGGGTTGCTGATGT-3^′^); CP2 (F: 5^′^-GTGAGTGACCCTGCTCCTTT-3^′^ and R: 5^′^-ACGGCATTCTTGGTTATTTG-3^′^); CP3 (F: 5^′^-GGCTTTATCAGGTCGGTGTA-3^′^ and R: 5^′^-TCTAGATGAACTACAACGCTCACCAG-3^′^). These three DNA fragments with overlapping sequences were individually inserted into the pMD18-T vector (Takara, http://www.takara.com.cn/) to produce the 7179 bp fragment. *Kpn* I- and *Xba* I-recognizing sequences were added to the ends. The 7179 bp fragment was digested with *Kpn* I and *Xba* I and inserted into the binary vector pCAMBIA2300 (CAMBIA, http://www.cambia.org/) to generate the resulting binary vector. The resulting binary and empty pCAMBIA2300 vectors were introduced into the *ygl138*-mediated transformation method (Liu et al. mutants using the *Agrobacterium tumefaciens*^2007^).

### RNA extraction and qRT-PCR

Total RNA was extracted using a TRIzol kit according to the manufacturer’s instructions (Invitrogen, http://www.invitrogen.com/). The RNA was pre-treated with DNase I and used for cDNA synthesis using an RT-PCR Kit (Promega, http://www.promega.com/). For qRT-PCR, SYBR Green I was added to the reaction system, and samples were run on a Chromo 4 real-time PCR detection system according to the manufacturer’s instructions (Bio-Rad, http://www.bio-rad.com/). The data were analyzed using Opticon monitor software (Bio-Rad). Each analysis was repeated three times. The rice *Actin1* gene was used as the internal control, and specific primers were: F: 5^′^-ACATCGCCCTGGACTATGACCA-3^′^ and R: 5^′^-GTCGTACTCAGCCTTGGCAAT-3^′^. The primers for qRT-PCR analysis of *YGL138*(*t*) expression were F: 5^′^-CTGGTCGCTGCAGATGTTTA-3^′^ and R: 5^′^-CTTTCTTTTGGCCTCTTCCA-3^′^.

## Electronic supplementary material

Additional file 1: Table S1: Markers used in mapping of *YGL138*(*t*). (DOC 42 KB)

Additional file 2: Figure S1: Sequence alignment of YGL138(t) protein of rice and its homologues proteins from other species. Identical residues are boxed in black, similar residues are high-lighted in gray. The N-terminal helical bundle domain and GTPase domain of YGL138(t) protein are underlined. Accession numbers of the protein sequences were as follows: *Oryza sativa* [*YGL138*(*t*), RAPDB: *Os11g05552*]; *Coccomyxa* sp. C-169 [GenBank: EIE18517]; *Nodularia spumigena* CCY9414 [GenBank: ZP_01629394]; *Actinomyces coleocanis* DSM 15436 [GenBank: ZP_03925628]; *Arabidopsis thaliana* [GenBank: NP_196014]; *Micromonas pusilla* CCMP1545 [GenBank: XP_003056150]; *Sorghum bicolor* [GenBank: XP_002442824]; *Zea mays* [GenBank: NP_001142003]. (TIFF 3 MB)

Below are the links to the authors’ original submitted files for images.

Authors’ original file for figure 1

Authors’ original file for figure 2

Authors’ original file for figure 3

Authors’ original file for figure 4

Authors’ original file for figure 5

Authors’ original file for figure 6

Authors’ original file for figure 7
