# Supplementary material for: YGL138(t), encoding a putative signal recognition particle 54 kDa protein, is involved in chloroplast development of rice
Source: Rice (N Y). 2013 Mar 27;6:7. doi: 10.1186/1939-8433-6-7 (PMC4883693; doi:10.1186/1939-8433-6-7)
Supplement: Supplementary file 1 — Additional file 1: Table S1: Markers used in mapping of YGL138(t). (DOC 42 KB) [file 12284_2012_43_MOESM1_ESM.doc]

**Additional file 1: Table S1.** Markers used in mapping of *YGL138*(*t*).

| Markers | Forward primer (5’→3’) | Reverse primer (5’→3’) | Loci (bp) |
| --- | --- | --- | --- |
| RM3668 | AGGAGGGAATCGTTCACAAG | GATCGTCTTCAACCTCCAGC | 1461169 |
| RM7557 | GTGTACTGCCATGAAAGGCC | GAAGTGCCTTTGCAGGAGAG | 2340664 |
| RM1812 | CAGCTAGTGAGCTCCTAGTG | GCTAACCCACCAACTTATTC | 2405238 |
| InDel-5 | TGCTGCGGAAGTCGCTGCTC | TCCGTCGGCCACCCAATCTC | 2497042 |
| RM6085 | GGTGAGAGATGGCTAAAGCG | CATCGCCTCTAGCACCTCC | 3041383 |
| RM5599 | CTCACAATATCACCATCCAC | AATTTTGTGCTGTTGTTGAA | 3824507 |
