# Supplementary figures and images for: YGL138(t), encoding a putative signal recognition particle 54 kDa protein, is involved in chloroplast development of rice
Source: Rice (N Y). 2013 Mar 27;6:7. doi: 10.1186/1939-8433-6-7 (PMC4883693; doi:10.1186/1939-8433-6-7)

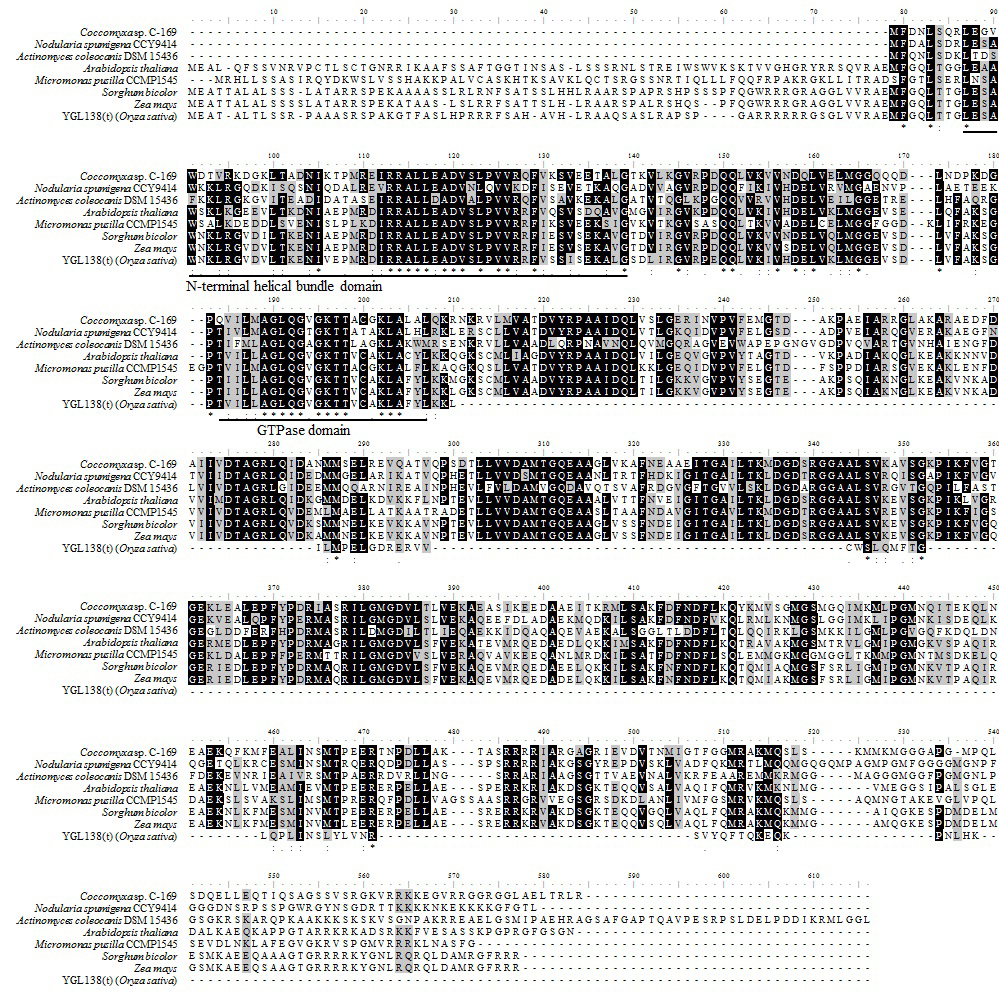

Supplement: Supplementary file 2 — Additional file 2: Figure S1: Sequence alignment of YGL138(t) protein of rice and its homologues proteins from other species. Identical residues are boxed in black, similar residues are high-lighted in gray. The N-terminal helical bundle domain and GTPase domain of YGL138(t) protein are underlined. Accession numbers of the protein sequences were as follows: Oryza sativa [YGL138(t), RAPDB: Os11g05552]; Coccomyxa sp. C-169 [GenBank: EIE18517]; Nodularia spumigena CCY9414 [GenBank: ZP_01629394]; Actinomyces coleocanis DSM 15436 [GenBank: ZP_03925628]; Arabidopsis thaliana [GenBank: NP_196014]; Micromonas pusilla CCMP1545 [GenBank: XP_003056150]; Sorghum bicolor [GenBank: XP_002442824]; Zea mays [GenBank: NP_001142003]. (TIFF 3 MB) [file 12284_2012_43_MOESM2_ESM.tiff]

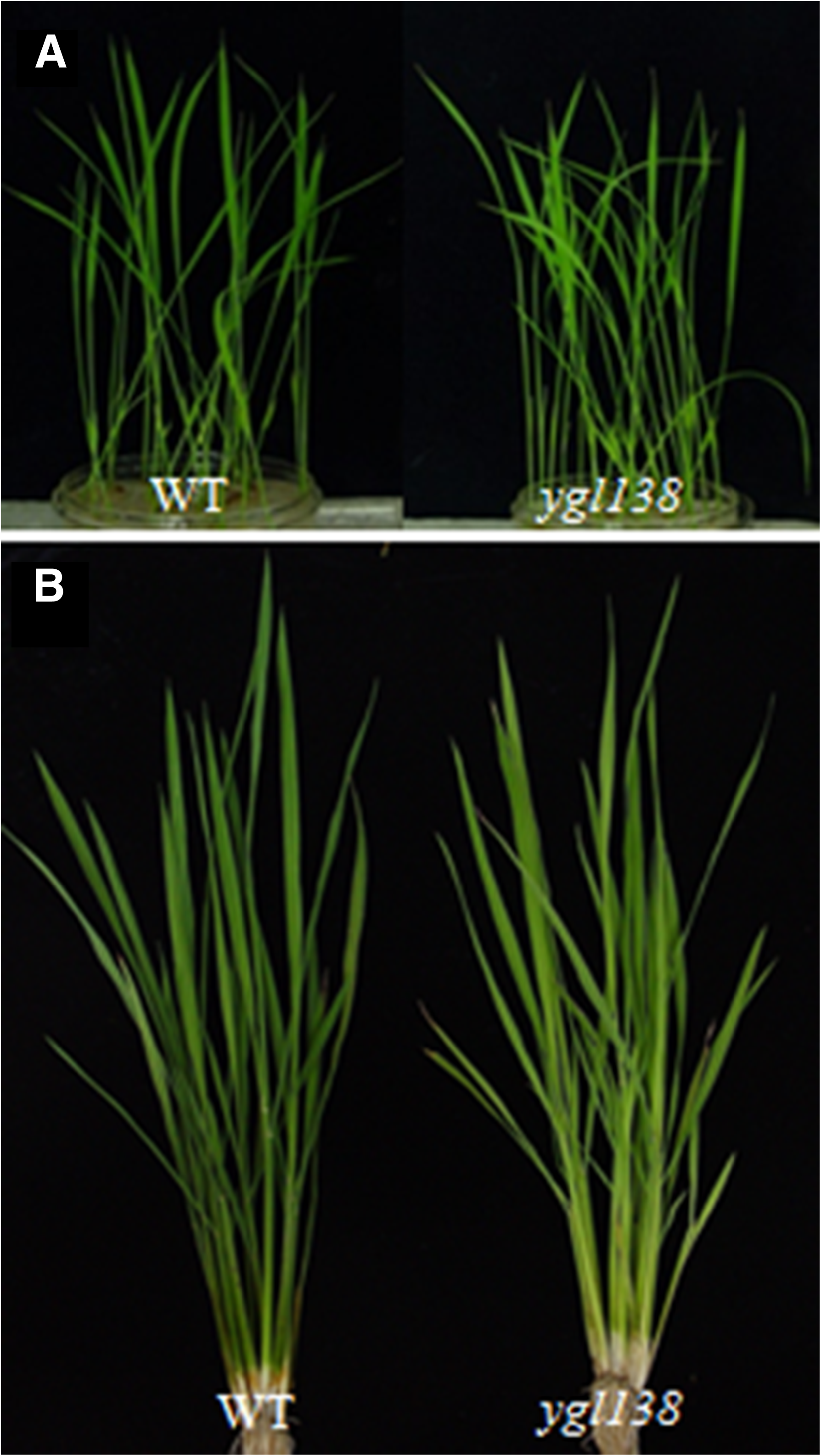

Supplement: Supplementary file 3 — Authors’ original file for figure 1 [file 12284_2012_43_MOESM3_ESM.tiff]

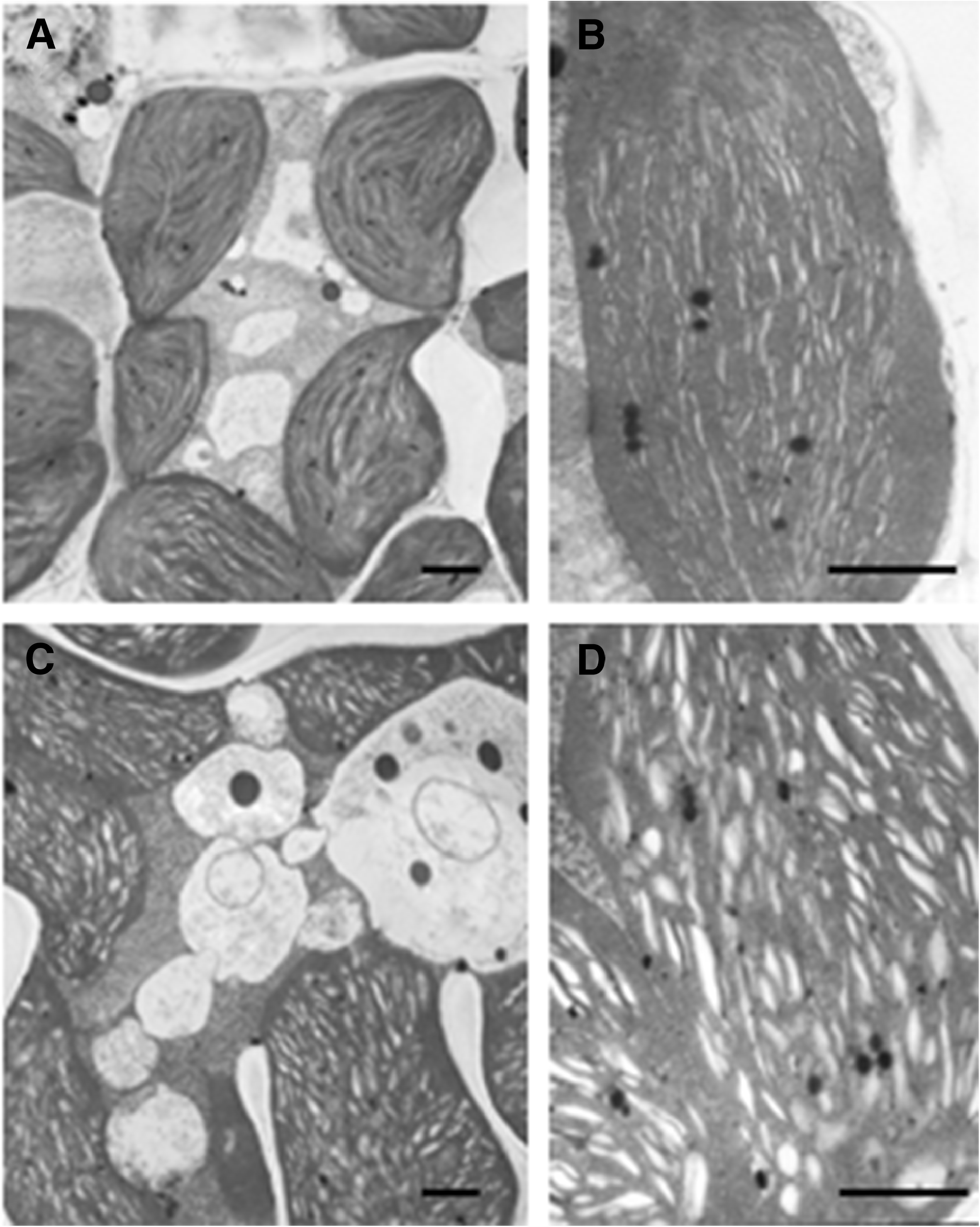

Supplement: Supplementary file 4 — Authors’ original file for figure 2 [file 12284_2012_43_MOESM4_ESM.tiff]

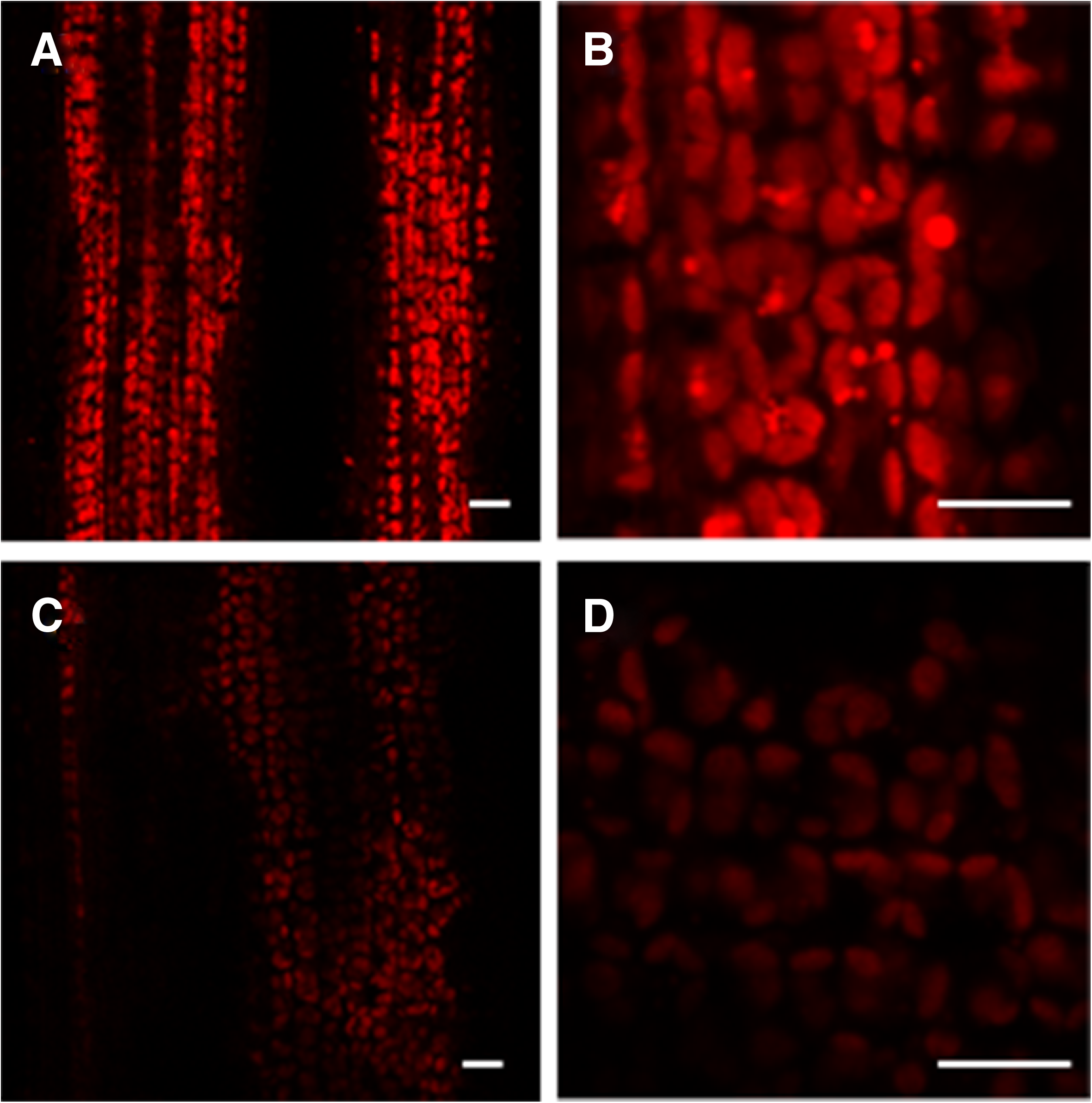

Supplement: Supplementary file 5 — Authors’ original file for figure 3 [file 12284_2012_43_MOESM5_ESM.tiff]

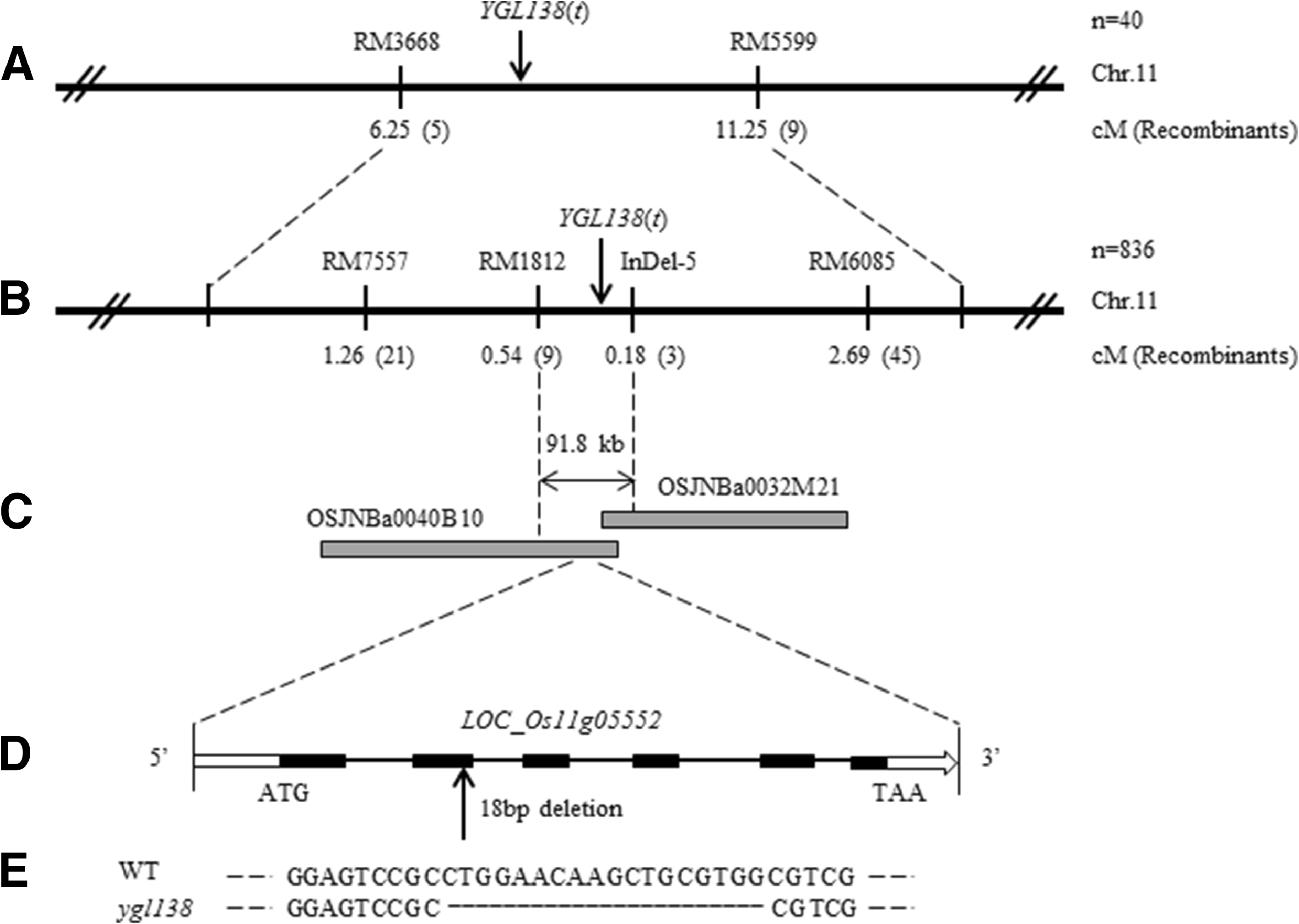

Supplement: Supplementary file 6 — Authors’ original file for figure 4 [file 12284_2012_43_MOESM6_ESM.tiff]

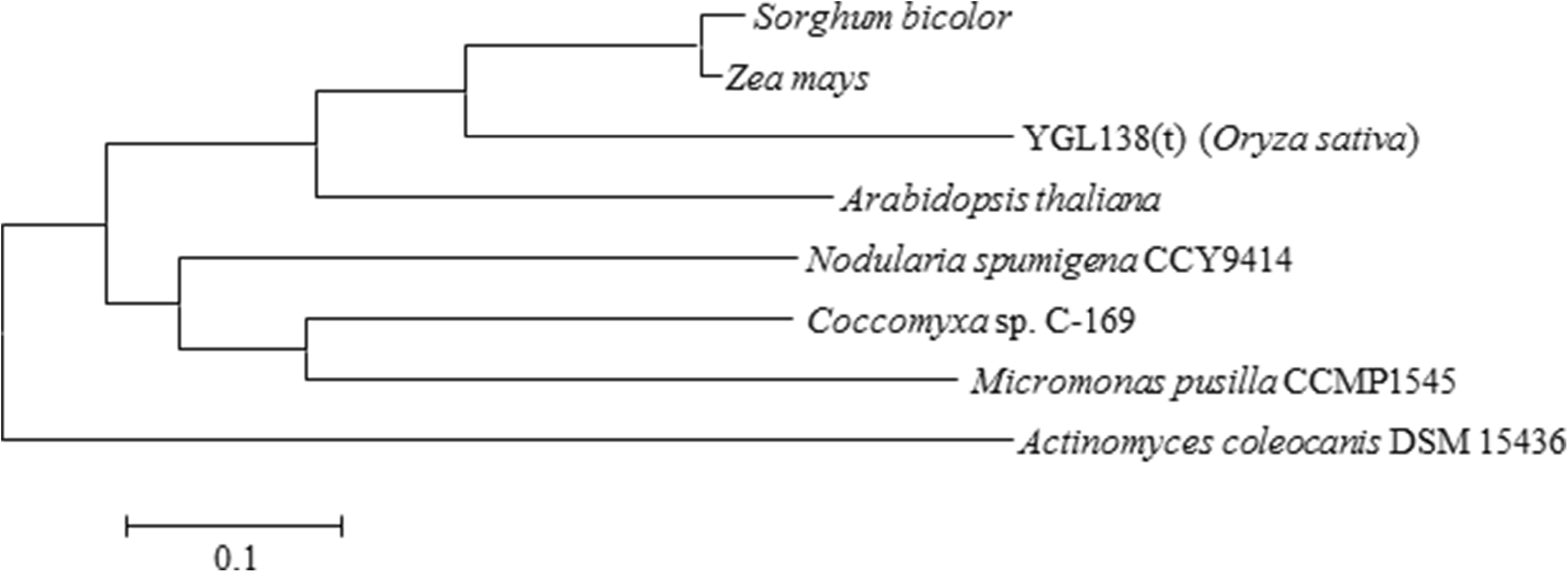

Supplement: Supplementary file 7 — Authors’ original file for figure 5 [file 12284_2012_43_MOESM7_ESM.tiff]

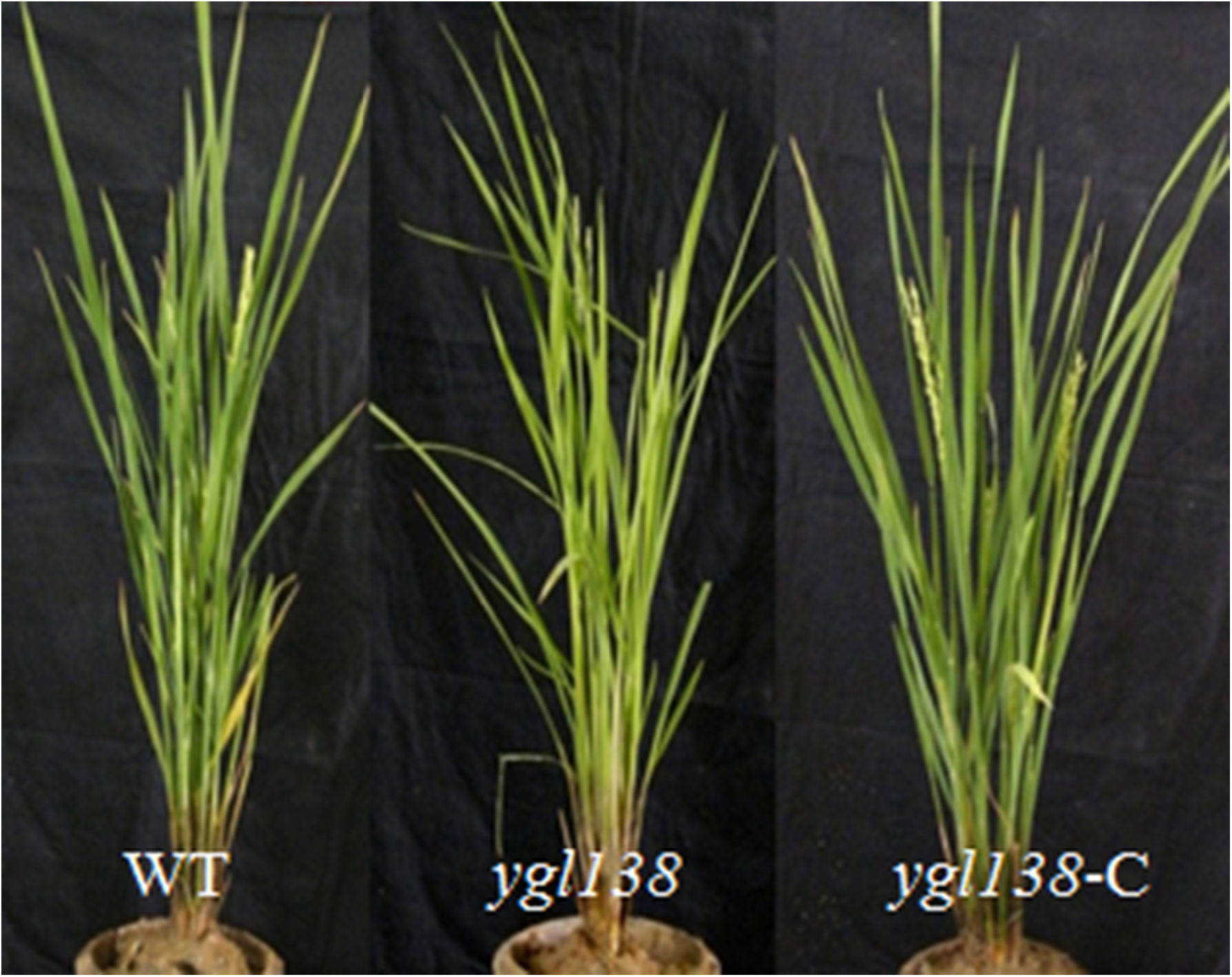

Supplement: Supplementary file 8 — Authors’ original file for figure 6 [file 12284_2012_43_MOESM8_ESM.tiff]

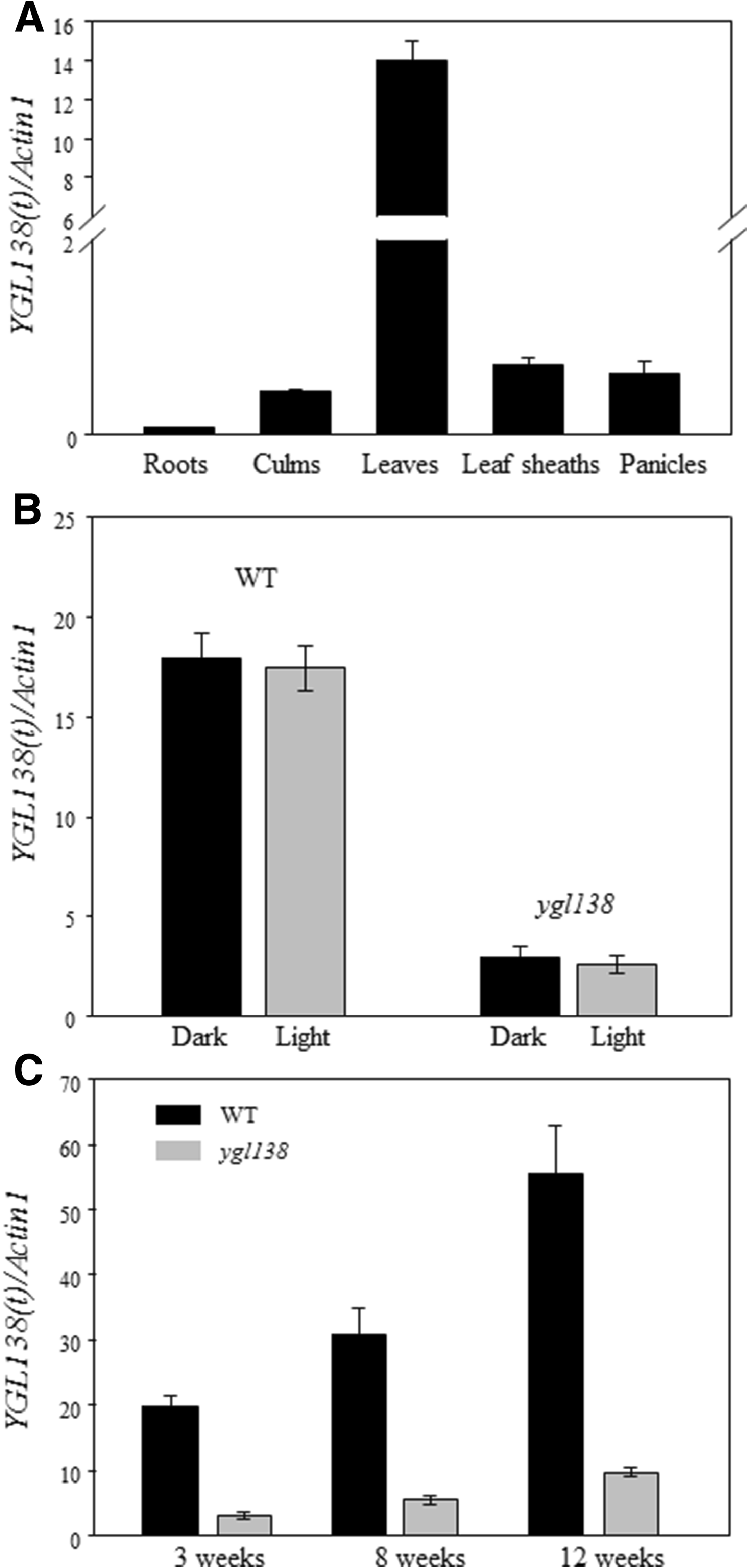

Supplement: Supplementary file 9 — Authors’ original file for figure 7 [file 12284_2012_43_MOESM9_ESM.tiff]
